# Excess cholesterol inhibits glucose‐stimulated fusion pore dynamics in insulin exocytosis

**DOI:** 10.1111/jcmm.13207

**Published:** 2017-05-25

**Authors:** Yingke Xu, Derek K. Toomre, Jonathan S. Bogan, Mingming Hao

**Affiliations:** ^1^ Department of Cell Biology Yale University School of Medicine New Haven CT USA; ^2^ Department of Bioengineering Key Laboratory of Biomedical Engineering of Ministry of Education Zhejiang University Hangzhou China; ^3^ Department of Internal Medicine Yale University School of Medicine New Haven CT USA; ^4^ Department of Biochemistry Weill Cornell Medical College New York NY USA

**Keywords:** insulin, exocytosis, cholesterol, fusion pore, total internal reflection fluorescence microscopy, beta cell, VAMP2‐pHluorin, diabetes, glucose

## Abstract

Type 2 diabetes is caused by defects in both insulin sensitivity and insulin secretion. Glucose triggers insulin secretion by causing exocytosis of insulin granules from pancreatic β‐cells. High circulating cholesterol levels and a diminished capacity of serum to remove cholesterol from β‐cells are observed in diabetic individuals. Both of these effects can lead to cholesterol accumulation in β‐cells and contribute to β‐cell dysfunction. However, the molecular mechanisms by which cholesterol accumulation impairs β‐cell function remain largely unknown. Here, we used total internal reflection fluorescence microscopy to address, at the single‐granule level, the role of cholesterol in regulating fusion pore dynamics during insulin exocytosis. We focused particularly on the effects of cholesterol overload, which is relevant to type 2 diabetes. We show that excess cholesterol reduced the number of glucose‐stimulated fusion events, and modulated the proportion of full fusion and kiss‐and‐run fusion events. Analysis of single exocytic events revealed distinct fusion kinetics, with more clustered and compound exocytosis observed in cholesterol‐overloaded β‐cells. We provide evidence for the involvement of the GTPase dynamin, which is regulated in part by cholesterol‐induced phosphatidylinositol 4,5‐bisphosphate enrichment in the plasma membrane, in the switch between full fusion and kiss‐and‐run fusion. Characterization of insulin exocytosis offers insights into the role that elevated cholesterol may play in the development of type 2 diabetes.

## Introduction

The clinical onset of type 2 diabetes mellitus (T2DM) is triggered by insufficient insulin secretion from pancreatic β‐cells to compensate for insulin resistance. Pancreatic β‐cell dysfunction plays an essential and critical role in the transition from pre‐diabetes to overt diabetes. Among many contributing factors, hyperlipidemia is a key element in the pathogenesis of β‐cell dysfunction 1. The diabetogenic effects of elevated circulating free fatty acids have been studied extensively. In addition to high free fatty acids, it has been reported that more than 70% of adults with T2DM have elevated cholesterol levels 2, 3. Diminished capacity of serum to remove cholesterol from pancreatic islets is also observed in diabetic patients 4, 5, 6. Evidence for a direct role of excess cholesterol in β‐cell dysfunction was first presented in 2007 7, 8. Islets from mice lacking the ABCA1 cholesterol transporter specifically in β‐cells have increased cholesterol and impaired glucose‐stimulated insulin secretion (GSIS) 7. Similarly, elevated islet cholesterol in ApoE‐deficient mice inhibited GSIS 8. Direct manipulation of membrane cholesterol in cultured β‐cells further showed that excess cholesterol impairs GSIS 8. Together, these data indicate that membrane cholesterol is involved in the regulation of GSIS. Since then, a close link between elevated cholesterol and impaired β‐cell function has been confirmed (reviewed in 9, 10). However, the molecular mechanisms of how cholesterol regulates GSIS have not been studied in detail. Because cholesterol is an essential determinant of membrane properties in mammalian cells, it is conceivable that the effects of cholesterol on GSIS are widespread across β‐cell function 10. Indeed, it has been shown that in β‐cells, cholesterol can act through plasma membrane (PM) potassium and calcium channels 11, 12, phosphatidylinositol 4,5‐bisphosphate (PIP_2_) 13, regulation of glucokinase activity by neuronal nitric oxide synthase 8 and insulin granule biosynthesis 14, 15.

Our previous study showed that overloading cholesterol enhances PM PIP_2_ and cortical F‐actin accumulation in cultured β‐cells 13. Because both PIP_2_ and cortical actin are intimately involved in regulating insulin granule dynamics at the cell surface, we investigated in this study the effect of excess cholesterol on insulin granule exocytosis. Regulation of fusion pore dynamics by cholesterol is important for insulin secretion, because premature closure of the fusion pore is a likely mechanism by which the release of insulin is prevented 16. The classic model of exocytosis describes full fusion, in which the exocytic vesicle and the PM mix completely. However, evidence exists for the presence of kiss‐and‐run events, in which insulin granules release their contents through a transiently opened fusion pore 17, 18, 19, 20. The relative occurrence of kiss‐and‐run events in β‐cells varies widely among reports, from a complete absence 21 to the vast majority 22. Whether β‐cells can generate differential release of nucleotides and peptides depending on their cellular cholesterol concentration is an intriguing question. While studies on insulin exocytosis have focused almost exclusively on proteins, the role of membrane lipids, with the exception of phosphoinositides, has yet to be incorporated in the understanding of the regulatory mechanism. More importantly, although numerous studies have used methods to reduce membrane cholesterol, few have looked at the effects of increased membrane cholesterol, which is more relevant to the pathophysiology of T2DM.

Traditional biochemical assays of exocytosis, such as radioimmunoassay or ELISA, have a limited temporal resolution. While methods such as capacitance measurements and carbon fibre amperometry have provided valuable insights into the mechanisms of exocytosis, they only report if exocytosis has occurred with limited information about the pre‐ and post‐exocytic events 23. Information about such events can be gained by the high spatiotemporal resolution imaging of individual granule fusion during exocytosis. Using fluorescent proteins targeted to the secretory granules and real‐time imaging of granule fusion in living cells by total internal reflection fluorescence microscopy (TIRFM), it is now possible to study insulin exocytosis in detail. TIRFM images have unsurpassed signal‐to‐noise ratio, virtually no out‐of‐focus fluorescence, and minimal photobleaching compared with other forms of imaging techniques 24. Dual‐colour TIRF microscopes with rapid switching and high synchronized frame rates allow for simultaneous imaging of two exocytic components, resulting in a more detailed characterization of processes at the PM. VAMP2‐pHluorin (or synapto‐pHluorin) is made up of a pH‐sensitive form of GFP fused to the luminal side of vesicle‐associated membrane protein 2 (VAMP2). Both endogenous VAMP2 and VAMP2‐GFP are localized to granules in β‐cells 25, 26, 27. When present in an intact granule, VAMP2‐pHluorin is non‐fluorescent due to the acidic pH in the lumen of insulin granules 28. Upon fusing with the PM, VAMP2‐pHluorin is exposed to the neutral extracellular buffer and displays an 11‐fold increase in its fluorescence 16. A fusion event is then detected as the appearance of a brightly fluorescent spot. The fact that VAMP2‐pHluorin is virtually non‐fluorescent before fusing with the PM dramatically reduces background fluorescence. Together with the fact that pH neutralization requires only a transiently open fusion pore, this property greatly increases the sensitivity and efficiency to detect fusion events with VAMP2‐pHluorin.

Using VAMP2‐pHluorin in this study, we characterized in detail the effect of cholesterol overloading on the fusion of insulin granules with the PM in cultured MIN6 insulinoma cells. We show that while the number of fusion events decreased with excess cholesterol, the fraction of kiss‐and‐run fusion, as well as clustered and compound exocytosis, was increased. We also present evidence that altered PIP_2_ and dynamin localization may contribute to the effect of excess cholesterol on insulin granule exocytosis.

## Materials and methods

MIN6 cultured β cells (passages 16–30) were grown in DMEM (11 mM glucose) supplemented with 100 U/ml penicillin, 100 μg/ml streptomycin, 10% FBS, 2 mM l‐glutamine and 50 μM 2‐mercaptoethanol. KRBH buffer (128.8 mM NaCl, 4.8 mM KCl, 1.2 mM KH_2_PO_4_, 1.2 mM MgSO_4_, 2.5 mM CaCl_2_, 5 mM NaHCO_3_, 10 mM HEPE, pH 7.4) was used for all experiments. Transient transfections were performed with Lipofectamine 2000 (ThermoFisher Scientific, Waltham, MA USA) according to the manufacturer's protocol, and cells were cultured for 48 hrs prior to microscopy. Reagents: VAMP2‐pHluorin (Dr. James Rothman, Yale University), PH_PLCδ_‐GFP and dynamin antibody (Dr. Pietro De Camilli, Yale University), insulin antibody (Cell Signaling). MβCD, water‐soluble cholesterol (CHOL) and all other chemicals were from Sigma.

### Cholesterol manipulation

For live‐cell imaging, cholesterol depletion using methyl‐β‐cyclodextrin (MβCD) was carried out by incubating cells with 10 mM MβCD at 37°C for 30 min.; to cholesterol overload, cells were incubated with 5 mM soluble cholesterol (1 g contains ∼40 mg cholesterol) at 37°C for 30 min. Cholesterol content was quantified in a 96‐well plate by a fluorometric method using an enzyme‐coupled reaction provided by the Amplex Red Cholesterol Assay kit (ThermoFisher Scientific), as described previously 8.

### Total internal reflection fluorescence microscopy

VAMP2‐pHluorin‐expressing MIN6 cells grown in MatTek dishes were first serum‐starved in 2 mM glucose for 2 hrs in KRBH. Cells were kept in an Air‐therm (WPI) temperature‐regulated environmental box at 37°C throughout the imaging experiments. Cells were imaged for 2 min. to establish basal baseline. A 10× concentrated stock of glucose was then added to the edge of the MatTek dish on the microscope stage. TIRFM was performed using an Olympus objective‐type IX‐70 inverted microscope fitted with a 60× /1.45 NA TIRFM lens (Olympus, Center Valley, PA, USA), controlled by Andor iQ software (Andor Technologies, South Windsor, CT, USA) and detected with a back‐illuminated Andor iXon 897 EMCCD camera (512  ×  512, 14 bit; Andor Technologies). The depth of the evanescent field was calculated to be 98 nm 29. Images were acquired without binning at 5–10 Hz in one channel or by sequential excitation at 3–5 Hz in two channels.

### Fluorescence microscopy

Confocal microscopy was performed using an Axiovert 100 M inverted microscope equipped with an LSM 510 laser‐scanning unit and a 63 × 1.2 NA plan Apochromat objective (Carl Zeiss, Inc., Thornwood, NY, USA). 488‐ and 543‐nm laser lines were used to excite the green and the red fluorophores, respectively. Emitted light was collected through band pass filters of 505–550 nm and 560–615 nm, respectively. For immunostaining, cells were fixed with 4% paraformaldehyde for 20 min., permeabilized with 0.1% Triton X‐100 for 5 min., blocked with 5% normal goat serum for 30 min., stained with primary antibodies for 1 hr and secondary antibodies for 30 min., all at room temperature.

### Live imaging of PH_PLCδ_‐GFP‐expressing cells

Images of MIN6 cells expressing PH_PLCδ_‐GFP were taken before and after glucose. A minimum laser power that would give a useful fluorescence signal was used, and the laser intensity was kept the same for all cells. Midcell sections were used to examine PH_PLCδ_‐GFP translocation. Serum‐starved cells were first imaged to establish a ‘basal’ image. A 10× concentrated stock was then added to the edge of the MatTek dish on the microscope stage for final concentrations of 20 mM glucose. The same cells were imaged again 15 min. after glucose addition. The microscope stage was kept at 37°C throughout treatment and imaging. Some cells were treated with 5 mM soluble cholesterol at 37°C for 30 min. before imaging. To quantify the ratio of PH_PLCδ_‐GFP fluorescence at the PM to that in the cytosol, two small regions that cover the PM and the cytosol (Cyto) were identified in the ‘basal’ image and also transferred to the ‘glucose’ image. The ratio of average fluorescence intensity of the two regions was calculated and denoted as I_PM_ */*I_Cyto_.

### Electron microscopy

Mouse pancreatic islets were isolated and cultured as described 14. Islets were fixed with 2.5% glutaraldehyde in 0.1 M sodium cacodylate buffer (pH 7.4) for 1 hr, rinsed with 0.1 M sodium cacodylate buffer and post‐fixed with 1% osmium tetroxide in 0.1 M cacodylate buffer for 1 hr, at room temperature. Samples then underwent a series of ethanol dehydration and embedded in EMbed812 resin, before sectioned (60 nm) and contrasted with 2% uranyl acetate and lead citrate. Images were taken on FEI Tecnai Biotwin transmission electron microscope using Morada CCD camera and iTEM software (Olympus).

### Image and statistical analysis

All image analysis used MetaMorph Image Analysis Software (Molecular Devices, Sunnyvale, CA, USA). 3D intensity profiles were generated in ImageJ. Analysis of fusion events was performed as described previously 29. Briefly, each time‐lapse movie was manually inspected to visually identify fusion events by the appearance of VAMP2‐pHluorin fluorescence. The coordinates and starting frame of a fusion event were marked. An ending frame was marked when the punctate spot of VAMP2‐pHluorin fluorescence was no longer distinguishable from the PM background. This occurred when VAMP2‐pHluorin either diffused into the PM *via* full fusion or dimmed away from the PM *via* kiss‐and‐run fusion. To perform intensity line scan in MetaMorph, a line was drawn across a horizontal montage of the first 10 frames generated from a small region of interest around a fusion event. For display purposes, some images were applied a low‐pass filter in MetaMorph to suppress noise. Unless otherwise indicated, data are presented as the mean ± S.E.M., and statistical significance analysed using a Student's *t*‐test.

## Results

### Cholesterol alteration changes the rate of insulin granule exocytosis

**Figure 1:**
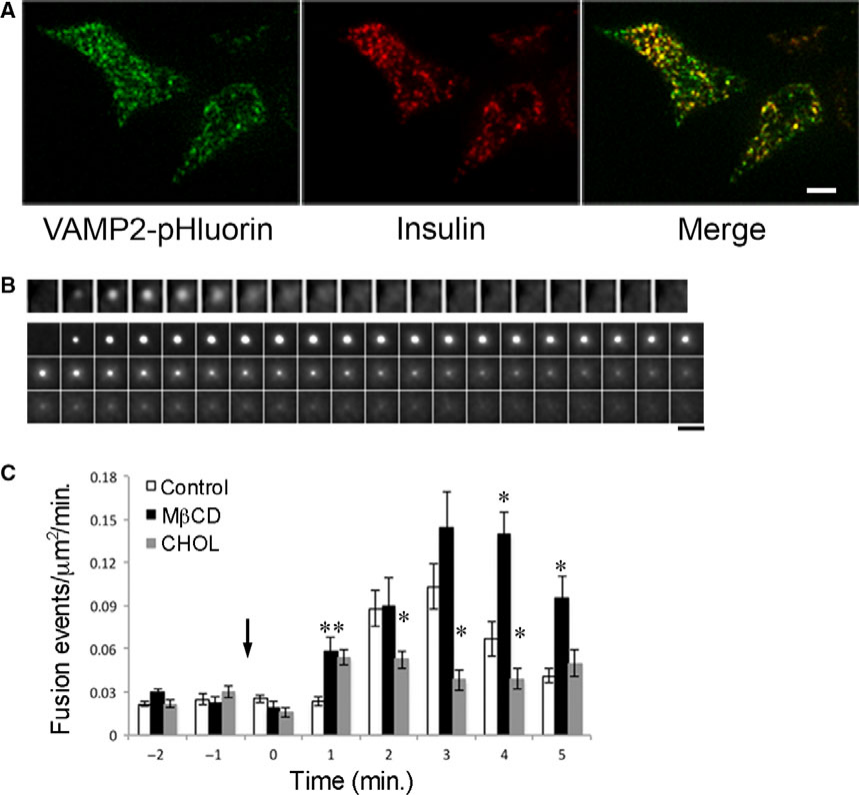
Excess cholesterol reduces insulin exocytosis. (**A**) VAMP2‐pHluorin is targeted to insulin granules in MIN6 cells. MIN6 cells expressing VAMP2‐pHluorin were fixed, permeabilized, stained with insulin antibody and imaged by TIRFM. Scale bar, 10 μm. (**B**) Glucose‐stimulated fusion events imaged by TIRFM. MIN6 cells were transfected with VAMP2‐pHluorin and stimulated with 20 mM glucose. Scale bar, 1 μm. (**C**) Cholesterol regulation of glucose‐stimulated insulin fusion events. MIN6 cells transfected with VAMP2‐pHluorin were pretreated with MβCD to deplete cholesterol or soluble cholesterol (CHOL) to overload cholesterol before stimulated with 20 mM glucose. Arrow points when glucose was added. *P < 0.05 *versus* Control. Control: 569 fusion events from 8 cells; MβCD: 317 fusion events from 3 cells; CHOL: 332 fusion events from 6 cells.

We used MIN6 insulinoma cell line expressing VAMP2‐pHluorin to investigate insulin granule exocytosis. Figure 1A shows a TIRFM image of VAMP2‐pHluorin, revealed by exposing the cells to neutral pH after permeabilization and immunostained with a monoclonal insulin antibody. A large portion of VAMP2‐pHluorin colocalized with insulin, consistent with previous finding that VAMP2 and fluorescently tagged VAMP2 are targeted to the insulin granules in β‐cells 22, 25, 27, 30. Figure 1B shows TIRFM images of glucose‐stimulated fusion of single granules labelled with VAMP2‐pHluorin in MIN6 cells. A sample time‐lapse video of two cells is included in Video S1. Stimulation of cells with 20 mM glucose caused fluorescent spots to suddenly appear, spread and diffuse laterally. Shown in the second panel is a prolonged fusion event, in which the fluorescence duration was >10 sec. and the signal did not spread in the PM before dimming and disappearing. Both examples in Figure 1B represent fusion events as opposed to perpendicular movement of a granule in and out of the evanescent field, because pHluorin became fluorescent only when the lumen of a granule came in contact with the extracellular buffer through the formation of a fusion pore. The short‐lived signals (first panel) could represent full fusion, in which VAMP2‐pHluorin fluorescence rapidly disappears due to lateral diffusion upon full dilation of the fusion pore. The long‐lived signals without spreading (second panel) could reflect the retrieval and slow re‐acidification of a granule in a kiss‐and‐run event.

Figure 1C addresses the question of whether cholesterol is involved in insulin granule exocytosis. Cells were incubated with 10 mM methyl‐β‐cyclodextrin (MβCD) and 5 mM soluble cholesterol at 37°C for 30 min. prior to TIRFM, which resulted in 38% ± 13% decrease and 71% ± 18% increase in cellular cholesterol levels, respectively, similar to previous studies 8, 13. Cells pretreated with MβCD to acutely deplete cellular cholesterol (Fig. 1C black bars) increased glucose‐stimulated insulin exocytosis compared with the control cells (Fig. 1C white bars) and cholesterol overloading significantly blunted glucose‐stimulated insulin exocytosis (Fig. 1C grey bars). These results are consistent with previous studies of GSIS using cell capacitance and insulin ELISA measurements from cells in which cholesterol was manipulated pharmacologically 8, 11. Because T2DM is often associated with obesity and elevated cholesterol contributes to β‐cell dysfunction, this study focused on the effect of increased cholesterol on insulin granule exocytosis.

### Two types of fusion events based on VAMP2‐pHluorin fluorescence profile

**Figure 2:**
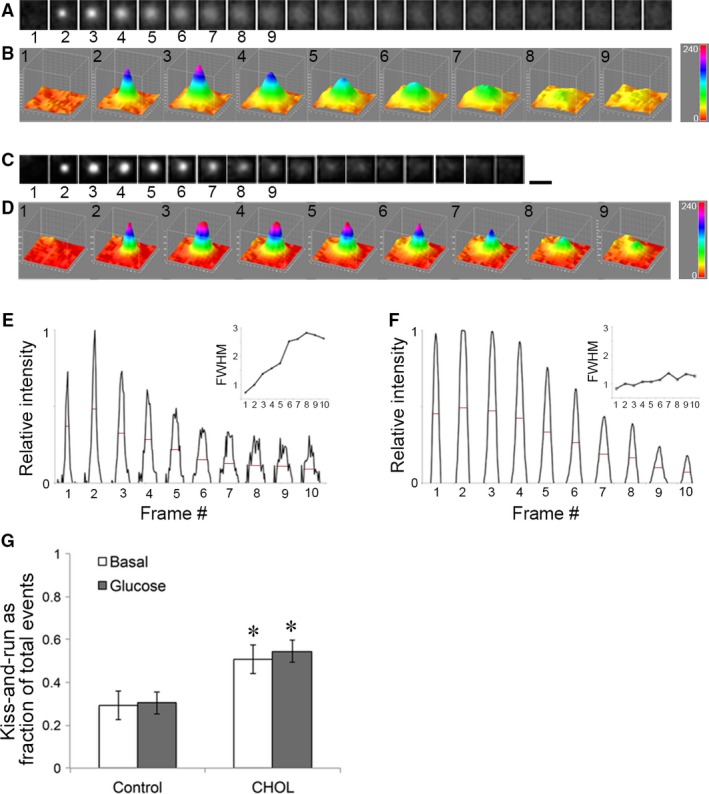
Identification of full and kiss‐and‐run fusion based on VAMP2‐pHluorin fluorescence profile. (**A**–**D**) Two examples of VAMP2‐pHluorin fusion events with different fluorescence profile of pore opening. (**A**,** B**) VAMP2‐pHluorin appeared upon fusion pore formation and spread laterally as the granule collapsed into the PM in a full fusion event. (**C**,** D**) VAMP2‐pHluorin appeared upon fusion pore formation and gradually dimmed without lateral spreading in a kiss‐and‐run fusion event. The 3D fusion profiles of selected frames are shown in B, D, with the colours representing pixel intensity indicated by the colour scale bar. Black scale bar, 1 μm, applies to A, C. (**E**,** F**) Line scans of the intensity profile from a full fusion event (**E**) and a kiss‐and‐run fusion event (**F**). Insets, fold change in full‐width‐at‐half‐maximum (FWHM, red lines) from the intensity line scans, normalized to that in frame 2. (**G**) The fraction of kiss‐and‐run events relative to total fusion events was quantified under basal and glucose‐stimulated conditions for both control and CHOL‐treated cells. *P < 0.05 *versus* Control.

The identification of full and kiss‐and‐run fusion events could be performed based on the fluorescence profile of the VAMP2‐pHluorin signal, a membrane protein associated with the insulin granule. Due to its sensitivity to pH, VAMP2‐pHluorin remained dark until a sudden flash upon fusion with the PM. Full fusion gave rise to the appearance of a ‘puff’ in which the fluorescence signal spread outward before disappearing when VAMP2‐pHluorin was fully integrated into the PM and diffused laterally (Fig. 2A). Disappearance of fluorescence signal without significant spreading represented kiss‐and‐run fusion (Fig. 2C). Full fusion could be represented by a continuous increase in the measured full‐width‐at‐half‐maximum from a 3D plot (Fig. 2B) or a line scan of the intensity profile (Fig. 2E red lines and inset). On the other hand, the VAMP2‐pHluorin signal in a kiss‐and‐run fusion event displayed a relatively stable width before disappearing from the PM (Fig. 2D, F and inset). Figure 2G shows that compared with untreated control cells, cholesterol overload (‘CHOL’) significantly increased the portion of kiss‐and‐run events.

### Two types of fusion events based on fusion pore duration

**Figure 3:**
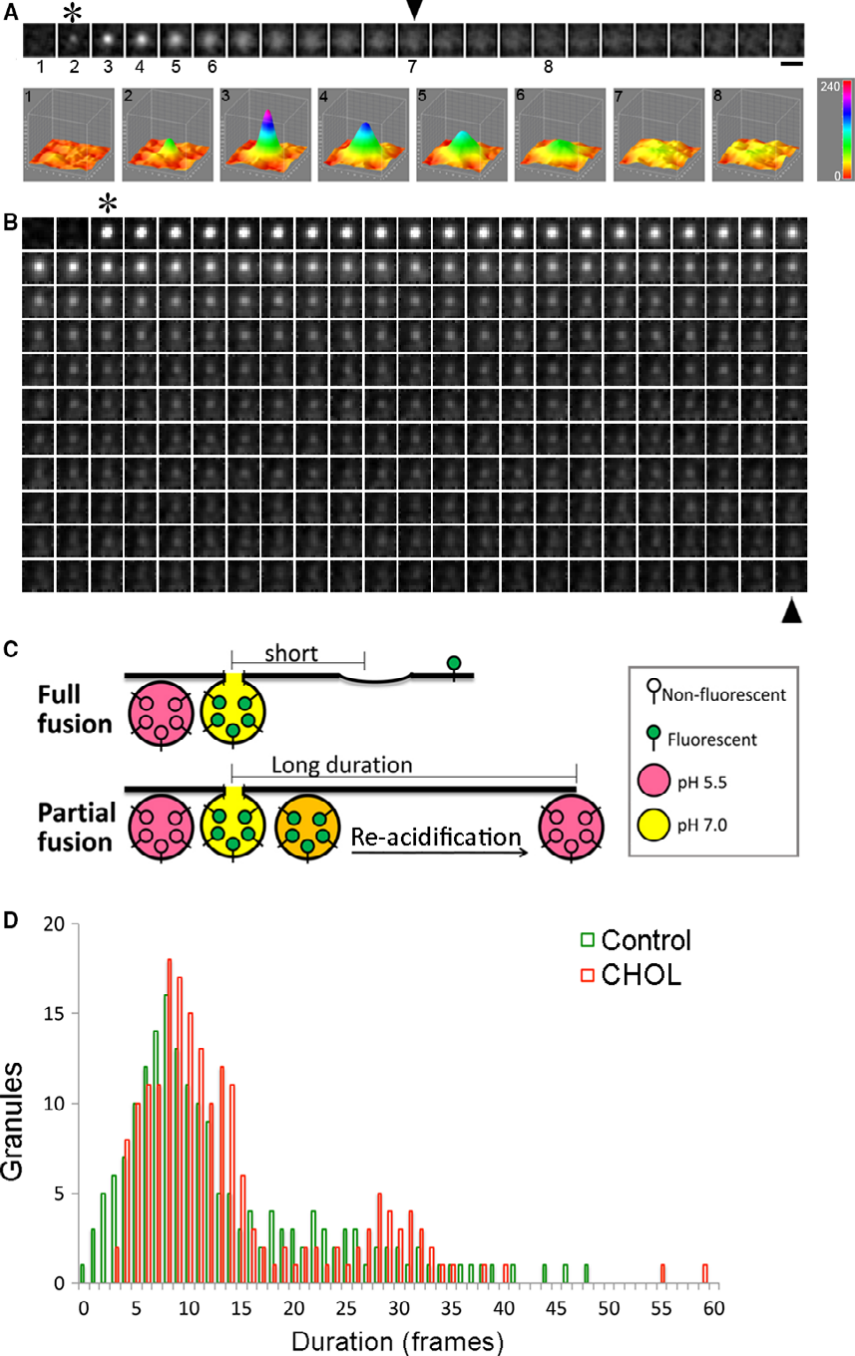
Identification of full and kiss‐and‐run fusion based on VAMP2‐pHluorin fluorescence duration. (**A**,** B**) VAMP2‐pHluorin appears (*) upon fusion pore formation. It disappeared (arrowhead) as the insulin granule collapsed into the PM in a full fusion event (**A**) or persists for a long time without spreading in a kiss‐and‐run fusion event (**B**). Scale bar, 1 μm, applies to A and B. (**C**) Schematic drawing of how the two types of fusion could give rise to short and long VAMP2‐pHluorin fluorescence duration. (**D**) Control MIN6 cells transfected with VAMP2‐pHluorin and ones pretreated with soluble cholesterol (CHOL) were stimulated with 20 mM glucose. Stimulated fusion events from 3 cells in each condition were plotted according to the amount of time a fluorescent spot remains distinctly visible after making its first appearance.

In addition to relying solely on the shape change of a fusion spot, we sought to supplement our morphological analysis with another quantification method. Using VAMP2‐pHluorin, we could identify the opening of a fusion pore by the first appearance of pHluorin fluorescence and full insulin granule collapse by the lateral spread of pHluorin fluorescence. This is demonstrated in Figure 3A by the frame in which the signal first appeared (*) and the frame in which the signal was no longer distinct from the PM (arrowhead). For a partial or kiss‐and‐run event, the majority of VAMP2‐pHluorin remained associated with the insulin granule membrane and did not merge into the PM (Fig. 3B). It has been shown that fusion events could be grouped into two populations according to the fluorescence duration of the VAMP2‐pHluorin signal, defined as the amount of time a fluorescent spot remains distinctly visible after making its first appearance 16, 31. In full fusion, the disappearance of VAMP2‐pHluorin fluorescence signal depends largely on the lateral diffusion of VAMP2‐pHluorin in the PM (Fig. 3C, top). In kiss‐and‐run fusion, the disappearance of VAMP2‐pHluorin fluorescence signal depends on re‐acidification of the insulin granule lumen after the fusion pore is sealed (Fig. 3C, bottom). Based on this rationale, the time course of fluorescence decrease seen in a long‐lived VAMP2‐pHluorin spot might indicate the time it takes for granule retrieval and re‐acidification after a kiss‐and‐run event, as suggested previously 32.

To see whether the two types of insulin fusion can be differentiated by the fluorescence duration of VAMP2‐pHluorin, a histogram of fusion events categorized by the duration of a fluorescence signal was plotted in Figure 3D. In both control and cholesterol‐overloaded (‘CHOL’) cells, there were two populations of events according to fluorescence duration. The two populations were more distinct in cholesterol‐overloaded cells. The simplest interpretation is that the first group corresponds to rapid full fusion events and the second one corresponds to kiss‐and‐run fusion with varying lengths of retrieval and re‐acidification time. This is consistent with previous classifications, in which VAMP2‐pHluorin signal with a rapid decay was interpreted as full fusion events, and kiss‐and‐run events were indicated by signals that remained punctuate and then dimmed due to re‐acidification of the granule 16, 31. The kiss‐and‐run fusion events (second population) increased in cholesterol‐overloaded cells (28% *versus* 39% for control and cholesterol‐overloaded cells, respectively, Fig. 3D). Because fluorescence duration depends on the time it takes to re‐acidify as well as how long a granule stays in the evanescent field, this type of measurement may underestimate the true proportion of kiss‐and‐run events.

### Excess cholesterol induces clustered fusion

**Figure 4:**
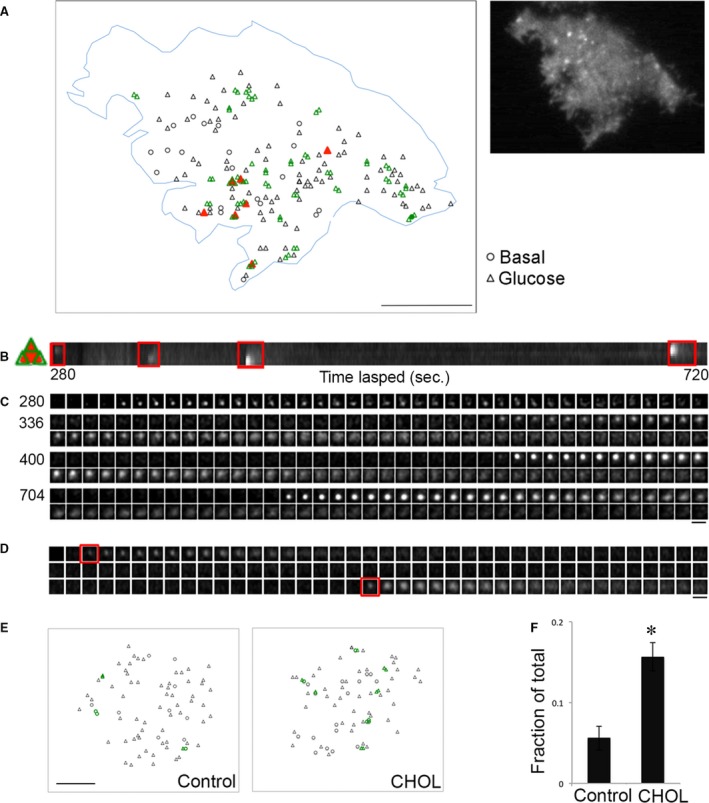
Spatial clustering of fusion events is increased by excess cholesterol. (**A**) All fusion events detected in a time‐lapse movie from a cholesterol‐overloaded cell were mapped, with circles labelling fusion at basal 2 mM glucose and triangles labelling fusion at stimulated 20 mM glucose. If the centre of a fusion event was within 2 pixels (378 nm) of another, it was coloured green; if the centre of a fusion event overlapped with another, it was coloured red. (**B**) Intensity kymograph in a 3 × 3 μm box around four fusion events. (**C**) Profiles of 4 fusion events occurring within a 3 × 3 μm box extracted from a continuous montage (Fig. S1). The numbers to the left refer to the time (sec.) after glucose was added. Intervals between frames were 200 ms. (**D**) An example profile of two fusion events whose centre coordinates are the same. Time‐lapse video is Video S2. (**E**) Side‐by‐side comparison of two cells having similar size and total number of fusion events, one control and one cholesterol overloaded (CHOL), with the fusion events mapped as in A. (**F**) Quantification of the fusion events present in a cluster defined in A, as a fraction of total fusion events. Control: *n* = 569 events from 8 cells; CHOL:* n* = 332 events from 6 cells. *P < 0.05. Scale bars: 5 μm for A and E, 1 μm for C and D.

As we probed through hundreds of fusion events, we noticed in cholesterol‐overloaded cells that there were regions in the PM that had repeated appearance of fusion events. To see whether there was spatial clustering for insulin granule exocytosis, we analysed the 2D distribution of fusion events in the PM. Clustered fusion was defined here as fusion events occurring at different times within one granule diameter on the PM (2 pixels or 378 nm). An example of the analysis of the spatial distribution of fusion events in a cholesterol‐overloaded cell is shown in Figure 4. Fusion events that were found within one granule diameter of each other were coloured green, and overlapping fusion events with the same set of coordinates were coloured red. Figure 4B shows the kymograph of a 3 × 3 μm region containing four fusion events shown in Figure 4A. These fusion events are further demonstrated individually in Figure 4C. A continuous montage from the time‐lapse video is shown in Figure S1. The numbers to the left indicate the time (sec.) after glucose was added. The clustered fusion events involved both kiss‐and‐run and full fusion (Fig. 4C). Figure 4D shows an example of two exocytic events taking place 19.8 sec. apart at the same site with superimposed coordinates. The time‐lapse movie for Figure 4D is included in Video S2. To show side by side the effect of excess cholesterol on inducing spatial clusters of fusion events, we chose two cells with similar areas and number of fusion events treated with or without soluble cholesterol (Fig. 4E). There was more clustered fusion, indicated by the increased green labelling in the ‘CHOL’ image to the right. As quantified in Figure 4F, cholesterol overloading resulted in a threefold increase in the fraction of the clustered fusion, defined as a fusion spot having its centre within two pixels (<400 nm) of the centre of another fusion spot during the first 10 min. after glucose stimulation. Clustered fusion sites are rarely observed for insulin granule exocytosis in normal β‐cells, where fusion events are typically well separated 33, similar to our observation in control cells. Thus, we conclude that the effect of excess cholesterol to cause clustering of fusion events indicates a true change in insulin granule exocytic properties.

### Excess cholesterol increases compound exocytosis

**Figure 5:**
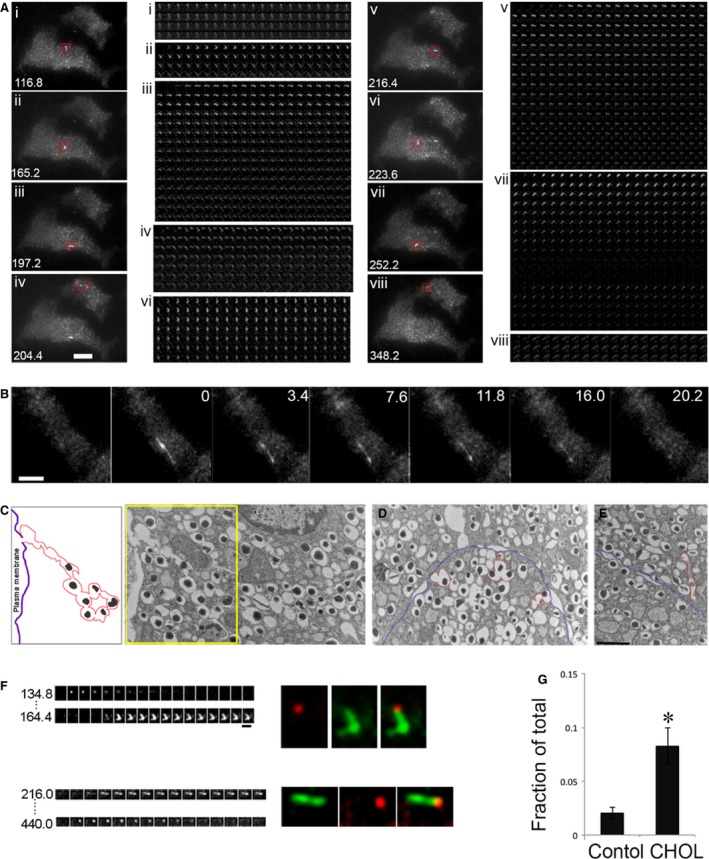
Compound exocytosis is increased by excess cholesterol. (**A**) Several examples of compound exocytosis from two cholesterol‐overloaded cells, leading to the appearance of fluorescent structures that were tubular instead of punctate in the PM. The number in the lower left corner is the time (sec.) after addition of glucose. The montages to the right for each exocytic event show that these structures were relatively stable and did not dilate like the single exocytic events. Time‐lapse video is Video S3. (**B**) An extreme case of compound exocytosis that became fluorescent all at once, and remained stable for >25 sec. Expanded montage is in Figure S2A and time‐lapse video is Video S4. (**C**–**E**) EM images of cholesterol‐overloaded islet cells, with multigranular structures outlined in red and the PM in purple. (**F**) Two examples of multiple fusion events at the same fusion pore. Red and green present single and compound exocytosis, respectively. Expanded montages are in Figure S2B. (**G**) Quantification of compound exocytosis, defined as having a tubule structure with length >2 μm, and expressed as a fraction of total fusion events. Control: *n* = 569 events from 8 cells; CHOL:* n* = 332 events from 6 cells. *P < 0.05. Scale bars: 5 μm for A and B**,** 1 μm for C‐F.

Exocytosis involving more than one vesicle is referred to as compound exocytosis 34. Figure 5A shows numerous tubular structures that appeared within 4 min. after glucose addition in two cholesterol‐overloaded cells during the time‐lapse movie (Video S3). Unlike single‐granule fusion, the structures in compound exocytosis were greater than 2 μm in length, remained fluorescent for tens of seconds and did not integrate into the PM (Fig. 5A montages). An example of an extremely elongated structure (approximately 8 μm) is shown in Figure 5B, Figure S2 and Video S4. We next turned to ultrastructural studies by electron microscopy (EM) for morphologic identification of compound exocytosis in cholesterol‐overloaded cells. The multigranular structure in Figure 5C resembled the ones shown by EM previously 35, 36. This example shows a chain of fused insulin granules with a pore opening at the PM. The insulin dense core content of the first several granules had presumably been released through the fusion pore. Granules connected to others without a complete membrane separation between the two neighbouring granules were included in the chain. We also observed granules that were fused together in a nonlinear fashion beneath the PM (Figs 5D and 3 events outlined in red), perhaps giving rise to the bent shapes upon fusion pore opening in Figure 5A. Some multigranular structures were found slightly away from the PM (Fig. 5E, red), which may represent granule–granule fusion prior to exocytosis or retrieval of such structures after an incomplete fusion (kiss‐and‐run) event. This may be the case in Figure 5A, panel vii. The tubular structure became fluorescent upon pH neutralization when the fusion pore opened transiently. It disappeared when it moved out of the evanescent field after fusion pore closing (middle of the montage), and reappeared when it moved back into the evanescent field. Although these single‐plane EM images only provide a partial representation of the fusion events, they are consistent with the presence of compound exocytosis in cholesterol‐overloaded cells.

Because we never observed compound exocytosis followed by full dilation of the fusion pore and lateral diffusion of VAMP2‐pHluorin in the PM, we suggest that the compound exocytic events induced by cholesterol overloading occur as kiss‐and‐run fusion events. The multigranular structures were not depleted after one round of exocytosis and may be prone to repeated fusion with the PM. This idea is supported by examples from two fusion sites in Figure 5A (panels ii and v), which are expanded in Figure 5F. In each site, two exocytic events took place at the same fusion pore opening (Fig. 5F, red and green). A continuous montage from the time‐lapse video is shown in Figure S2. Finally, we quantified the number of tubular structures that appeared upon fusion as a fraction of total fusion events. These data show that cholesterol overloading induced a fourfold increase in compound exocytosis (Fig. 5G).

### Dynamin is selectively associated with kiss‐and‐run fusion

**Figure 6:**
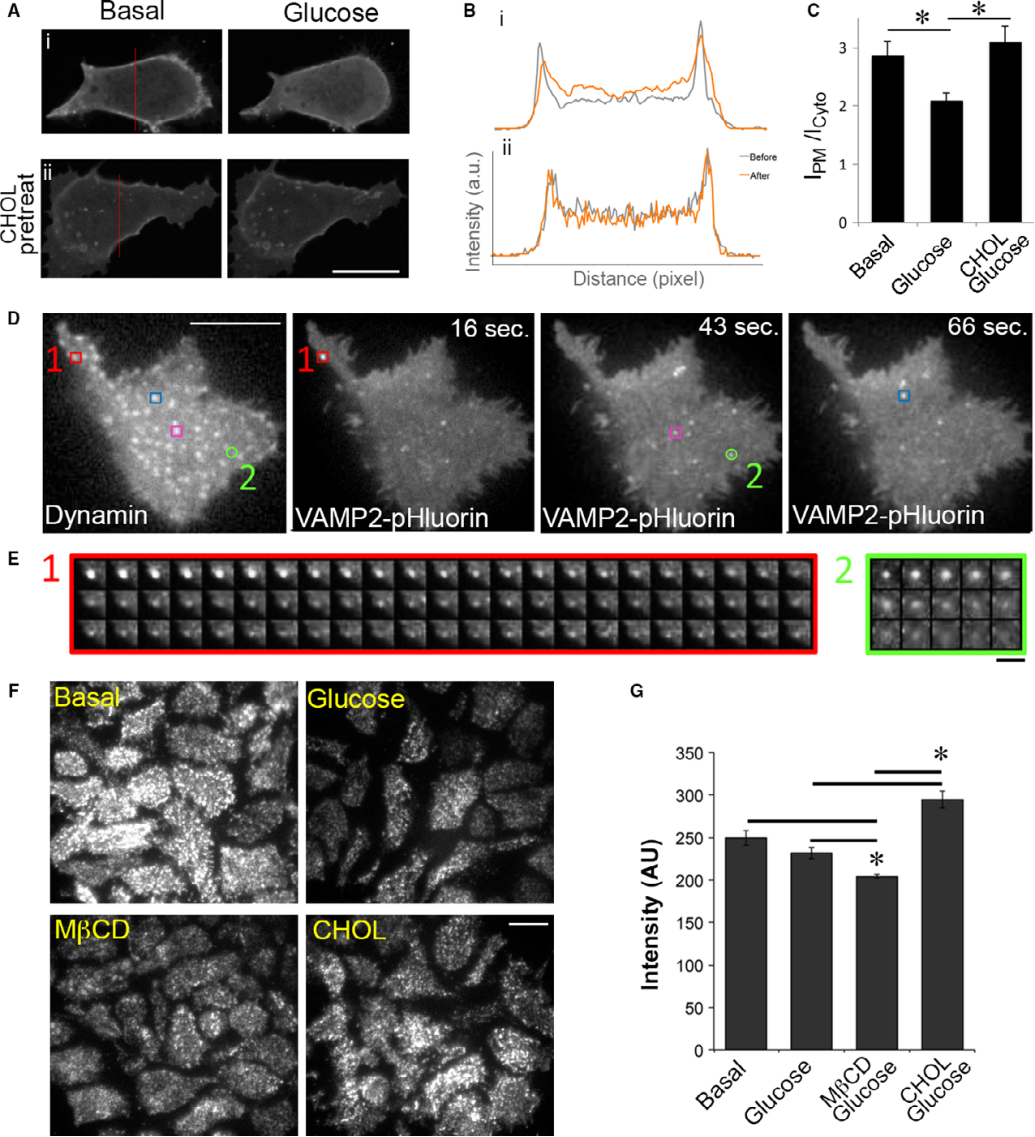
Dynamin is selectively associated with kiss‐and‐run fusion. (**A**) Excess cholesterol inhibits glucose‐mediated redistribution of PIP_2_. Mid‐cell sections were used to examine PH_PLC__δ_‐GFP translocation. Serum‐starved MIN6 cells were first imaged, and a 10× concentrated stock of glucose was then added to the cells on the microscope stage for 15 min. A final concentration of 20 mM glucose was used. Cells were pretreated with 5 mM CHOL for 30 min. prior to imaging in panel ii. (**B**) Corresponding fluorescence intensity line scan was plotted for images in A before (grey) and 15 min. after (orange) glucose. Cells were pretreated with 5 mM CHOL for 30 min. prior to imaging in panel ii. (**C**) Quantification for the ratio of PH_PLC__δ_‐GFP fluorescence at the PM (I_PM_) to that in the cytosol (I_C__yto_) as described in the Methods. Data show mean ± S.E.M.,* n* = 6 cells. * *P* < 0.05 by Student's *t*‐test. (**D**) MIN6 cells expressing dynamin‐mRFP and VAMP2‐pHluorin were stimulated with 20 mM glucose and imaged by dual‐colour TIRFM. The time after glucose addition is shown in the upper right corner. Three examples of fusion sites that contained dynamin (red, blue and purple squares) and one that did not contain dynamin (green circle) are shown here. (**E**) Montages of two fusion events represented by the red square (#1) and green circle (#2) in D show kiss‐and‐run and full fusion, respectively. (**F**,** G**) TIRFM imaging (**F**) and quantification (**G**) of dynamin within 100 nm of the PM. ‘Basal’ cells were starved in 2 mM glucose for 2 hrs, ‘glucose’ cells were incubated with 20 mM glucose for 15 min., ‘MβCD’ cells were incubated with 5 mM MβCD at 37°C for 1 hr, and ‘CHOL’ cells were incubated with 5 mM CHOL for 1 hr at 37°C. 20 mM glucose was added to the last 15 min. of cholesterol treatment. Cells were then fixed and stained with dynamin antibody. Eighteen imaging fields from three independent experiments were used for each condition. *P < 0.05 *versus* glucose, and *versus* MβCD. Scale bars: 10 μm for A, D and F**,** 1 μm for E.

Phosphatidylinositol 4,5‐bisphosphate (PIP_2_) plays an important role in GSIS. Glucose stimulates PIP_2_ hydrolysis to generate the signalling molecules diacylglycerol and inositol trisphosphate 37. The distribution and turnover of PIP_2_ can be assessed using a reporter containing GFP fused to the PH domain of PLCδ (PH_PLCδ_‐GFP) (Fig. 6A) 38. Quantification of fluorescence intensity from mid‐cell confocal images (Fig. 6A, panel i) revealed that glucose decreased PH_PLCδ_‐GFP in the PM and increased its abundance in the cytosol, indicating glucose‐stimulated PIP_2_ hydrolysis (Fig. 6B, panel i and 6C). However, when cells were pretreated with soluble cholesterol (CHOL), glucose was unable to stimulate PIP_2_ hydrolysis (compare Fig. 6B, i, ii and 6C). Increased PIP_2_ can activate the GTPase dynamin by a direct interaction 39. Dynamin has been shown to regulate the dynamics of exocytosis 26, 40, 41. Because dynamin can associate with vesicles at the time of fusion and inhibition of dynamin's GTPase activity has a threefold increase in full fusion events 42, we tested whether dynamin may play a role in cholesterol‐induced kiss‐and‐run fusion events.

MIN6 cells expressing dynamin‐mRFP and VAMP2‐pHluorin were stimulated with 20 mM glucose and imaged by dual‐colour TIRFM. Dynamin was observed at insulin fusion sites indicated by VAMP2‐pHluorin. Approximately 21% of fusion sites contained dynamin. Three examples of fusion sites that contained dynamin (red, blue and purple squares) and one that did not contain dynamin (green circle) are shown in Figure 6D. The distribution of dynamin did not change throughout the acquisition period; thus, only the first frame is displayed here. The VAMP2‐pHluorin profiles over time of fusion event #1 (red square, containing dynamin) and fusion event #2 (green circle, lacking dynamin) are shown in Figure 6E. Interestingly, fusion events at sites that contained dynamin (red, blue and purple squares) show characteristics of kiss‐and‐run fusion, in which the pHluorin signal decreased gradually over a long period of time without spreading (Fig. 6E, #1), whereas fusion spots that did not contain dynamin (green circle) displayed full fusion, in which signals spread (5th frame) and disappeared rapidly (Fig. 6E, #2, green circle lacking dynamin). In fact, out of the 147 events examined, none of the full fusion events was found to be associated with dynamin. These observations support the idea that dynamin participates in kiss‐and‐run, but not full fusion, exocytic events.

Our detailed analysis of the localization of dynamin to single fusion events corroborated the finding that dynamin content is correlated with the proportion of cellular kiss‐and‐run events 42. We next examined whether cholesterol overloading increased dynamin at the PM in β‐cells by immunofluorescence staining of dynamin in fixed cells. TIRFM shows cholesterol was involved in the regulation of dynamin accumulation near the PM of β‐cells. Cholesterol depletion decreased dynamin, while cholesterol overloading increased dynamin (Fig. 6F and G). The correlation among excess cholesterol, accumulation of PIP_2_ and dynamin recruitment (Fig. 6) suggests that excess cholesterol may shift full fusion to kiss‐and‐run fusion by recruitment of dynamin *via* PIP_2_.

## Discussion

The final step in insulin secretion is the fusion of insulin granules with the PM. The relative contribution of partial (kiss‐and‐run) fusion, and even its very existence, in insulin exocytosis is the subject of heated debate. Because cholesterol regulates PIP_2_ at the PM in β‐cells 13 and PIP_2_ mediates fusion pore formation 43, we propose that cholesterol may be directly involved in modulating fusion pore dynamics during glucose‐stimulated insulin exocytosis. The challenge in studying fusion pore has always been to distinguish unambiguously the two types of fusion. Thus, accurate identification of fusion type is key to studying fusion pore dynamics.

Biochemical measurements of insulin secretion showed that GSIS is reduced in β‐cells overloaded with cholesterol 7, 8. Because cholesterol is ubiquitously present in all cell membranes, the effect of cholesterol changes cannot be isolated to the plasma membrane or exocytosis. Using pH‐sensitive VAMP2‐pHluorin with TIRFM, we examined the mechanisms of cholesterol‐regulated insulin exocytosis by direct visualization of real‐time exocytic events from individual insulin granules. We show that cholesterol loading decreased fusion activity at the single‐granule level. Two types of fusion could be distinguished based on the fluorescence profile of VAMP2‐pHluorin (Fig. 2). In addition, the histograms in Figure 3 show that there were two distinct populations of fusion events according to VAMP2‐pHluorin fluorescence duration. We suggest that the short‐lived signals were full fusion, in which VAMP2‐pHluorin fluorescence rapidly disappeared due to lateral diffusion in the PM. The long‐lived signals represented kiss‐and‐run type of fusion, in which the fluorescence disappearance would reflect both the time for retrieval and the time required to acidify the granule lumen. Cholesterol may affect fusion pore dilation or protein diffusion in the PM, resulting in persistence of VAMP2‐pHluorin signal. However, fusion pore dilation and protein diffusion occur on a much faster time scale than granule acidification, making it unlikely that the puncta with extremely long fluorescence duration (tens of seconds) can be accounted for by slower fusion pore dilation or protein diffusion. The relative proportion of the two types of fusion shifted towards more kiss‐and‐run events in cholesterol‐overloaded β‐cells (Figs 2 and 3). The suppression of kiss‐and‐run events is essential to the appropriate insulin secretion in response to glucose stimulation in β‐cells. As such, dysregulation of kiss‐and‐run events may contribute to β‐cell dysfunction in cholesterol‐overloaded cells and possibly in T2DM.

Compound exocytosis is rarely observed in healthy β‐cells 44. We show that compound exocytosis, in which multiple granules fused together prior to forming a single exocytic fusion pore in the PM, was significantly increased in cholesterol‐overloaded cells. The formation of a dense actin network or membrane microdomains induced by excess cholesterol may account for the fusion and fission of multiple insulin granules in the vicinity of the same fusion site, leading to compound exocytosis. One potential mechanism for the formation of multigranular structures in compound exocytosis is redistribution of SNARE proteins from the PM into a fused granule 44. Cholesterol‐enriched lipid microdomains have been proposed to serve as platforms to assemble SNARE complexes. It is therefore possible that upon a kiss‐and‐run event, a granule may retrieve more concentrated SNARE proteins, which could increase subsequent fusion with other incoming granules to form multigranular structures. Dysregulation of compound exocytosis may lead to unregulated basal insulin secretion.

By regulating PIP_2_ in the PM, cholesterol can also alter the downstream effects of PIP_2_ 45. In control β‐cells, dynamin was present at a small portion of fusion sites. Cholesterol overloading induced dynamin accumulation at the PM. The presence of dynamin correlated with longer fluorescence durations and with a ‘no spread’ profile for the VAMP2‐pHluorin signals, consistent with kiss‐and‐run fusion (Fig. 6). Indeed, none of the full fusion events was found to be associated with dynamin. In cholesterol‐overloaded β‐cells, our data indicated that PIP_2_ accumulated and dynamin was recruited at the PM, so that kiss‐and‐run and compound exocytic events were favoured. This is consistent with the finding that retrieval of secretory granules depends on dynamin 40. A small molecule activator of dynamin, Ryngo 1‐23, was shown to reduce the fraction of kiss‐and‐run events in chromaffin cells 46, suggesting that the effect of excess cholesterol on fusion pore dynamics in β‐cells may be separate from dynamin activation. Our work and that of others 26, 40 show that the recruitment of dynamin is associated with kiss‐and‐run fusion. Because dynamin is regulated by direct interactions with actin 47, and excess cholesterol promotes PIP_2_‐medicated cortical actin polymerization 13, we propose that cholesterol‐induced stabilization of PIP_2_ and F‐actin leads to recruitment of dynamin, which increases kiss‐and‐run in cholesterol‐overloaded β‐cells. Together, our data support the idea that excess cholesterol may reduce overall insulin exocytosis by a dynamin‐dependent process that is activated by PIP_2_.

## Conflicts of interest

The authors confirm that there are no conflicts of interest.

## Supporting information


**Fig. S1** Continuous montage of the fusion events in Figure 4C.

**Fig. S2** Continuous montage of the fusion events in Figure 5B (A) and F (B).

**Video S1** Time‐lapse video of MIN6 cells transfected with VAMP2‐pHluorin and stimulated with 20 mM glucose. Frames were collected by TIRFM at 5 Hz and are shown at 30 frames per second. This video was highly compressed from the original 20 MB time‐lapse video.

**Video S2** Time‐lapse video of cholesterol‐overloaded MIN6 cells expressing VAMP2‐pHluorin and stimulated with 20 mM glucose. This video corresponding to Figure 4D shows an example of two exocytic events taking place 19.8 sec. apart with superimposed coordinates. Frames were collected by TIRFM at 5 Hz and are shown at 30 frames per second. This video was compressed from the original 6 MB time‐lapse video.

**Video S3** Time‐lapse video of cholesterol‐overloaded MIN6 cells expressing VAMP2‐pHluorin and stimulated with 20 mM glucose. This video shows the examples of multigranular structures displayed in Figure 5A. Frames were collected by TIRFM at 5 Hz and are shown at 60 frames per second. This video was highly compressed from the original 57MB time‐lapse video.

**Video S4** Time‐lapse video of cholesterol‐overloaded MIN6 cells expressing VAMP2‐pHluorin and stimulated with glucose. Highlighted here is the sudden appearance and elongation of a tubule‐shaped multigranular structure displayed in Figure 5B. Frames were collected by TIRFM at 5 Hz and are shown at 30 frames per second. This video was compressed from the original 5MB time‐lapse video.
