# Supplementary figures and images for: Excess cholesterol inhibits glucose‐stimulated fusion pore dynamics in insulin exocytosis
Source: J Cell Mol Med. 2017 May 25;21(11):2950–62. doi: 10.1111/jcmm.13207 (PMC5661106; doi:10.1111/jcmm.13207)

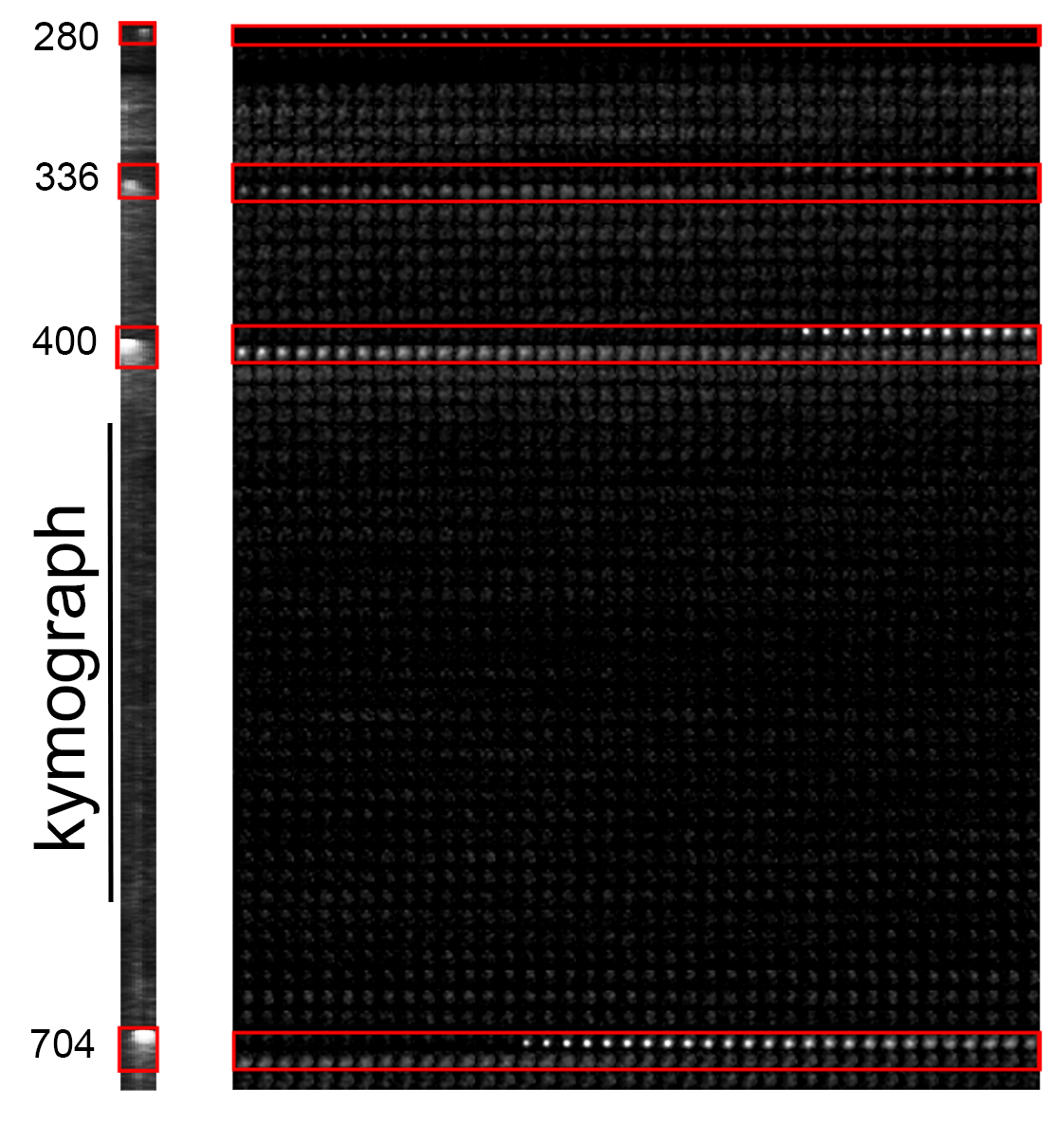

Supplement: Supplementary file 1 — Fig. S1 Continuous montage of the fusion events in Figure 4C. [file JCMM-21-2950-s001.tif]

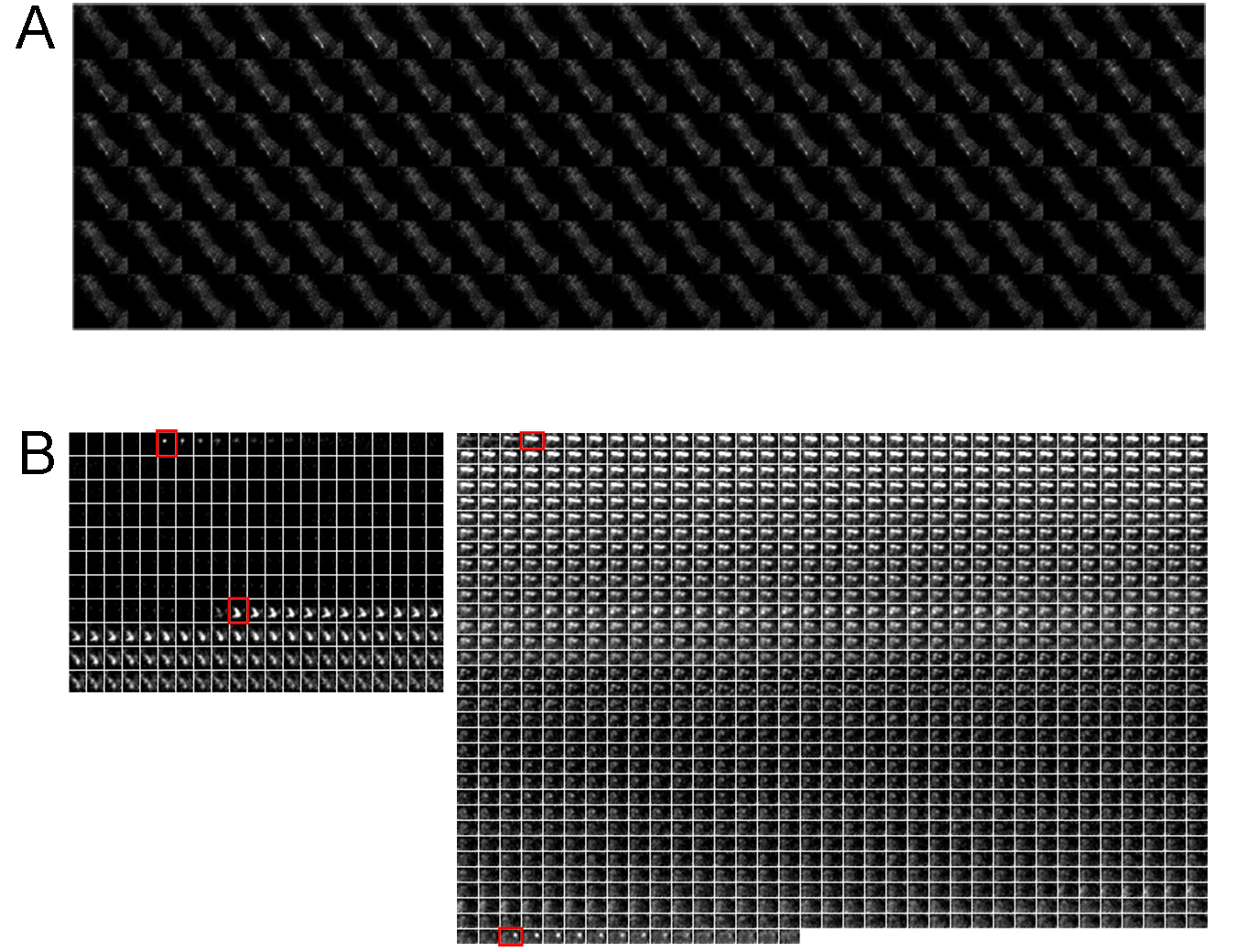

Supplement: Supplementary file 2 — Fig. S2 Continuous montage of the fusion events in Figure 5B (A) and F (B). [file JCMM-21-2950-s002.tif]
